# MicroRNA-93/STAT3 signalling pathway mediates retinal microglial activation and protects retinal ganglion cells in an acute ocular hypertension model

**DOI:** 10.1038/s41419-020-03337-5

**Published:** 2021-01-04

**Authors:** Yayi Wang, Shida Chen, Jiawei Wang, Yaoming Liu, Yang Chen, Tao Wen, Xiuli Fang, Manuel Vidal-Sanz, Jost B. Jonas, Xiulan Zhang

**Affiliations:** 1 Zhongshan Ophthalmic Center, State Key Laboratory of Ophthalmology, Sun Yat-Sen University, Guangzhou, 510060 China; 2 Eye Center of Shandong University, The Second Hospital of Shandong University, Jinan, 250100 China; 3 Departamento de Oftalmología, Facultad de Medicina, Universidad de Murcia, 30120 El Palmar, Murcia Spain; 4 Department of Ophthalmology, Medical Faculty Mannheim, Heidelberg University, Mannheim, 68167 Germany

**Keywords:** Microglia, Inflammation

## Abstract

Glaucoma is a common neurodegenerative disease and a leading cause of irreversible blindness worldwide. Retinal microglia-mediated neuroinflammation is involved in the process of optic nerve damage, but the mechanisms driving this microglial activation remain mostly elusive. Previous investigations reported that microRNAs are associated with the retinal microglial reaction and neural apoptosis. In the present study, we found that microRNA-93-5p (miR-93) played a key role in the reaction of retinal microglial cells in vivo and in vitro. The miR-93 level was significantly reduced in the retinae of rat acute ocular hypertension (AOH) models, which were accompanied by retinal microglial activation, overproduction of inflammatory cytokines, and subsequent retinal ganglion cells (RGCs) death, versus the retinae of controls. The induction of miR-93 overexpression significantly reduced microglial proliferation, migration and cytokine release, inhibited the expression of the target gene signal transducer and activator of transcription 3 (STAT3) and p-STAT3, and was associated with a reduced loss of RGCs. Treatment with a STAT3 inhibitor also decreased retinal microglial activation after AOH injury. Taken together, these results suggest that the miR-93/STAT3 pathway is directly related to the downregulation of retinal microglia-mediated neuro-inflammation and showed a neuroprotective effect. Regulating microglial activation through miR-93 may serve as a target for neuroprotective therapy in pathological ocular hypertension.

## Introduction

Primary angle-closure glaucoma is one of the main types of glaucoma. Elevated intraocular pressure (IOP) leads to glaucomatous optic neuropathy and irreversible optic nerve damage and blindness. The complex pathophysiology of IOP-induced optic nerve damage, however, has not been fully elucidated. Mounting evidence suggests that retinal microglia-mediated neuroinflammation plays a role in the glaucomatous optic nerve injury process^1,2^. Retinal microglial cells are the resident phagocytes which perform innate immune functions and maintain tissue homoeostasis. They are involved in almost all retinal pathological processes including retinal ganglion cell (RGC) damage and death in glaucoma. Previous studies have revealed that retinal microglia cells are significantly activated both in patients with glaucoma and in experimental animal models of glaucoma^3–7^. Influencing retinal microglial activation may thus be an important step in future neuroprotective treatments for glaucoma.

MicroRNAs (miRNAs) are evolutionarily conserved small non-coding RNAs that target messenger RNAs to repress their translation into proteins, playing a critical role in post-transcriptional regulation^8,9^. Previous studies have found that miRNAs expression significantly changed in glaucomatous eyes^10–12^. Moreover, accumulating evidence has demonstrated that miRNAs are important regulatory factors of microglial activation and polarisation in diseases of the central nervous system (CNS), and in retinal diseases such as age-related macular degeneration and diabetic retinopathy^13–17^. The results of these investigations have opened new avenues for miRNA-related microglia-targeted therapy for glaucoma.

Our previous study revealed that microRNA-93-5p (miR-93) was significantly suppressed in the aqueous humour of glaucoma patients and in the retinae of rodents with experimental glaucoma^12,18^. Bioinformatic analysis revealed that the target gene was associated with microglia-mediated neuro-inflammation and RGCs apoptosis. In addition, miR-93 has been reported to inhibit inflammatory cytokine production in lipopolysaccharide-stimulated macrophages^19^. Therefore, we explored the possible effects of miR-93 in modulating microglial reactions, inhibiting neuroinflammation, and protecting RGCs against acute ocular hypertension (AOH) in in vivo and in vitro experiments.

## Materials and methods

### Establishment of the AOH model

This study was approved by the Committee of Animal Care of the Sun Yat-Sen University. All animal experiments were performed in accordance with the Association for Research in Vision and Ophthalmology’s Statement for Use of Animals. Adult female Sprague–Dawley (SD) rats weighing 200–250 g were divided into five groups using a random number table: the control group, AOH group, agomir negative control group with AOH (NC + AOH), miR-93 agomir group with AOH (miR-93+AOH) and S3I-201 group with AOH (S3I + AOH). A signal transducer and activator of transcription 3 (STAT3) inhibitor, S3I-201 (2 mg/kg, Sigma-Aldrich, St. Louis, MO, USA), was intraperitoneally administered to rats just before AOH and on post injury day 2. Two microlitres of miR-93 agomir (a chemically modified miRNA agonist) or nontargeting NC (0.5 nmol/µl, Ribobio, Guangzhou, China) were injected intravitreally into the right eye with a microinjector 6 h before AOH induction. The miR-93 sequences were 5′-CAAAGUGCUGUUCGUGCAGGUAG-3′ and 3′-GUUUCACGACAAGCACGUCCAUC-5′; the NC sequences were 5′-UCACAACCUCCUAGAAAGAGUAGA-3′ and 3′-AGUGUUGGAGGAUCUUUCUCAUCU-5′. The intravitreal injection and AOH techniques were described in detail previously^3,18,20^. Briefly, a 30-gauge infusion needle was inserted into the anterior chamber of the right eye under anaesthesia and connected to a 250-ml sterile saline bag lifted to a height of 150 cm above the level of the eye. The IOP was measured using a TONOLAB tonometer (Icare^®^ TONOLAB, Tampere, Finland). After the IOP was maintained at an elevated level for 1 hour, the needle was withdrawn so that the IOP gradually dropped. After 3 days, all animals were sacrificed. All eyes were included in the study unless the establishment of the animal model was not successful, for example, rat died unexpectedly, AOH was not successfully induced, or lens was impaired. Separate interventional studies had been done regarding different evaluation objectives. For each group, at least three animals were applied.

### Immunohistochemistry and TUNEL staining

Following a previously described protocol, cryostat sections of whole eyes were analysed by immunohistochemistry with a microglia-specific IBA1 antibody (Cat# 019-19741, Wako, Osaka, Japan)^3^. Terminal deoxynucleotidyl transferase dUTP nick end labeling (TUNEL) staining was performed according to the manufacturer’s protocol (Cat# 12156792910, Roche, Mannheim, Germany). Six images were randomly captured from each retina for analysis. The microglial cells were defined as IBA1^+^ cells with an identifiable 4′,6-diamidino-2-phenylindole (DAPI)-stained nucleus and the apoptotic cells were identified by TUNEL^+^ cells with a DAPI-stained nucleus.

### Quantitative real-time polymerase chain reaction (qRT-PCR)

The retinae were freshly dissected and immediately frozen in liquid nitrogen. Total retinal RNA was extracted using a miRNeasy Mini Kit (QIAGEN, Hilden, Germany) according to the manufacturer’s instructions. MiR-93 reverse transcription primers (stem-loop) and qPCR primers (forward and reverse) were designed by RiboBio. Other primers included tumour necrosis factor (TNF)-α forward (fwd) 5′-ACCATGAGCACGGAAAGCAT-3′ and reverse (rev) 5′-AACTGATGAGAGGGAGCCCA-3′, interleukin (IL)-1β fwd 5′-TACCTATGTCTTGCCCGTGG-3′ and rev 5′-TAGCAGGTCGTCATCATCCC-3′, IL-6 fwd 5′-TCTGGTCTTCTGGAGTTCCGT-3′ and rev 5′-CTTGGTCCTTAGCCACTCCT-3′, STAT3 fwd 5′-TCGGAAAGTATTGTCGCCCC-3′ and rev 5′-GGACATCGGCAGGTCAATGG-3′, and glyceraldehyde-3-phosphate dehydrogenase (GAPDH) fwd 5’-AGTGCCAGCCTCGTCTCATA and rev 5′-TGAACTTGCCGTGGGTAGAG-3′. MiR-93 reverse transcription was performed with EZBioscience^®^ 4× microRNA Reverse Transcription Mix (EZBioscience, Roseville, MN, USA), and inflammatory cytokines and STAT3 were reverse transcribed using the PrimeScript^™^ RT Reagent Kit with gDNA Eraser (TAKARA Biotechnology, Shiga, Japan). qRT-PCR was performed using a commercial TB Green^®^ *Premix Ex Taq*^™^ II Kit (TAKARA Biotechnology) with a LightCycler^®^ 480 Instrument II (Roche, Basle, Switzerland). The PCR procedures consisted of 95 °C for 30 s followed by 50 cycles of 95 °C for 5 s and 60 °C for 30 s. U6 and GAPDH were used as endogenous controls. The data were calculated using the 2 − ΔΔCT method, and each sample was measured three times.

### Western blot analysis

Western blotting was performed as described previously^3^. Briefly, retinal proteins were separated on an sodium dodecyl sulfate-polyacrylamide gel and transferred to a polyvinylidene fluoride membrane (Bio-Rad, Hercules, CA, USA). The membrane was incubated with primary antibodies against STAT3 (1:400, Cat# sc-8019, Santa Cruz Biotech, Dallas, TX, USA), p-STAT3 (1:1000, Cat# 9145, Cell Signalling Technology, Danvers, MA, USA) and β-actin (1:200, Cat# sc-47778, Santa Cruz Biotech) overnight and then with horseradish peroxidase (HRP)-conjugated secondary antibody (Cell Signalling Technology). The bands were visualised with Immobilon Western Chemilum HRP Substrate (Millipore, Darmstadt, Germany) and an image capture system (Bio-Rad). Relative protein expression levels were measured by calculating the band density with ImageJ software (National Institutes of Health, USA).

### Cell culture

Primary retinal microglial cultures were prepared according to a detailed procedure described elsewhere^15^. Retinal tissues were separated from 3-day-old SD rats and mechanically dissociated into a single-cell suspension. The cells were then seeded in 25-cm^2^ cell culture flasks. The cells were kept in a humidified atmosphere of 5% CO_2_ and 95% air for 2 weeks. Subsequently, the microglial cells were harvested by shaking the flasks at 200 rpm for 2 h. The detached cells were collected by centrifugation and replated at designated densities for various experiments. Microglial cells were identified by immunocytochemical staining with an anti-IBA1 antibody (Cat# 019-19741, Wako). The cells were placed on sterile coverslips in 12-well plates and cultured for 5 days. After being fixed and blocked, the cells were incubated overnight with the primary antibody, washed in phosphate-buffered saline (PBS), and then incubated in an Alexa Fluor^®^ 488 Conjugate secondary antibody (Cell Signalling Technology). Images were collected under a fluorescent microscope (Zeiss LSM 510 Meta, Oberkochen, Germany).

### Transfection and oxygen–glucose deprivation (OGD)

Microglial cells were seeded in 12-well plates and divided into 4 groups: the control group, 1-h OGD and 24-h reperfusion (OGD1 h/R24 h) group, miR-93 mimic transfection group with OGD1 h/R24 h (miR-93 mimic + OGD1 h/R24 h), and miR-93 mimic NC transfection group with OGD1h/R24h (miR-93 NC + OGD1 h/R24 h). Then, 50 nM of miR-93 mimic and miR-93 NC (Thermo Fisher, Waltham, MA, USA) were transfected into cells using Lipofectamine 2000 Transfection Reagent (Invitrogen, Carlsbad, CA, USA) following the manufacturer’s instructions for 24 h. After that, microglial cells were cultured in glucose and serum-free DMEM (Gibco, Paisley, UK), exposed to a gas mixture of 94% N_2_/5% CO_2_/1% O_2_ at 37 °C for 1 h, and then returned to basal DMEM/F12 + 20% foetal bovine serum (FBS, Gibco) in a normoxic chamber for 24 h. Control cells were cultured under normal conditions for the same amount of time.

### Dual-luciferase reporter assay

Microglial cells were seeded in 24-well plates 24 h prior to transfection. The STAT3 3′-untranslated region (3′-UTR) harbouring the wild-type or mutant sequence of the miR-93 binding sites was cloned into pGL3-luc plasmids (Promega, Madison, WI, USA). The plasmids were co-transfected with the miR-93 mimic, mimic NC, miR-93 inhibitor or inhibitor NC (Ribobio) along with the pRL-TK Renilla plasmid cultured with microglia cells. The cells were harvested 36 h later, and the Dual-Luciferase Reporter Assay System (Promega) was used to detect the relative luciferase intensity.

### Evaluation of microglial activation

To evaluate the migration of microglial cells, the scratch wound assay and transwell assay were carried out. Cells were cultured until they reached 80% confluence and were scratched with a sterile tip. According to the different groups, the cells were transfected and exposed to OGD1 h/R24 h. Then, the cells were washed with PBS and fixed with 4% paraformaldehyde, and five independent fields were photographed to calculate the migration index based on the following formula: migration index = the number of migrating cells in the experimental group/control × 100%. Transwell chambers containing 8-µm pore filters (Corning Inc., Corning, NY, USA) were selected. After transfection and OGD1 h/R24 h, microglial cells were washed with PBS and inoculated at a density of 2 × 10^6^ in the upper chamber with 200 µl serum-free medium. The lower chamber was filled with 600 µl of DMEM medium supplemented with 10% FBS. After 12 h of incubation at 37 °C, the cells that migrated to the lower surface of the membrane were stained with 0.1% crystal violet for 30 min. Five random fields were photographed, and the cells were counted to estimate microglial migration. Furthermore, to evaluate microglial cell proliferation, immunocytochemistry for Ki-67 (Cat# ab16667, Abcam, Cambridge, UK) was performed using the same method described above.

### Measurement of inflammatory cytokine levels

After transfection and OGD1 h/R24 h, the culture medium was collected to measure the levels of TNF-α and IL-1β released by microglia using enzyme-linked immunosorbent assay kits (R&D Systems, Minneapolis, MN, USA) according to the manufacturer’s protocols.

### Statistical analysis

For in vitro experiments, we used three independent cultured cells per group. The experiments were independently repeated three times. The data are presented as the mean ± s.d. and were analysed using IBM SPSS Statistics for Windows, version 25.0 (IBM Corp., USA). Student’s *t* test was performed, and *P* value was selected from *t* test when the variance was not equal. Two-sided *P* values less than 0.05 were accepted as statistically significant.

## Results

### AOH downregulated miR-93, stimulated retinal microglial activation and caused RGCs death

**Fig. 1:**
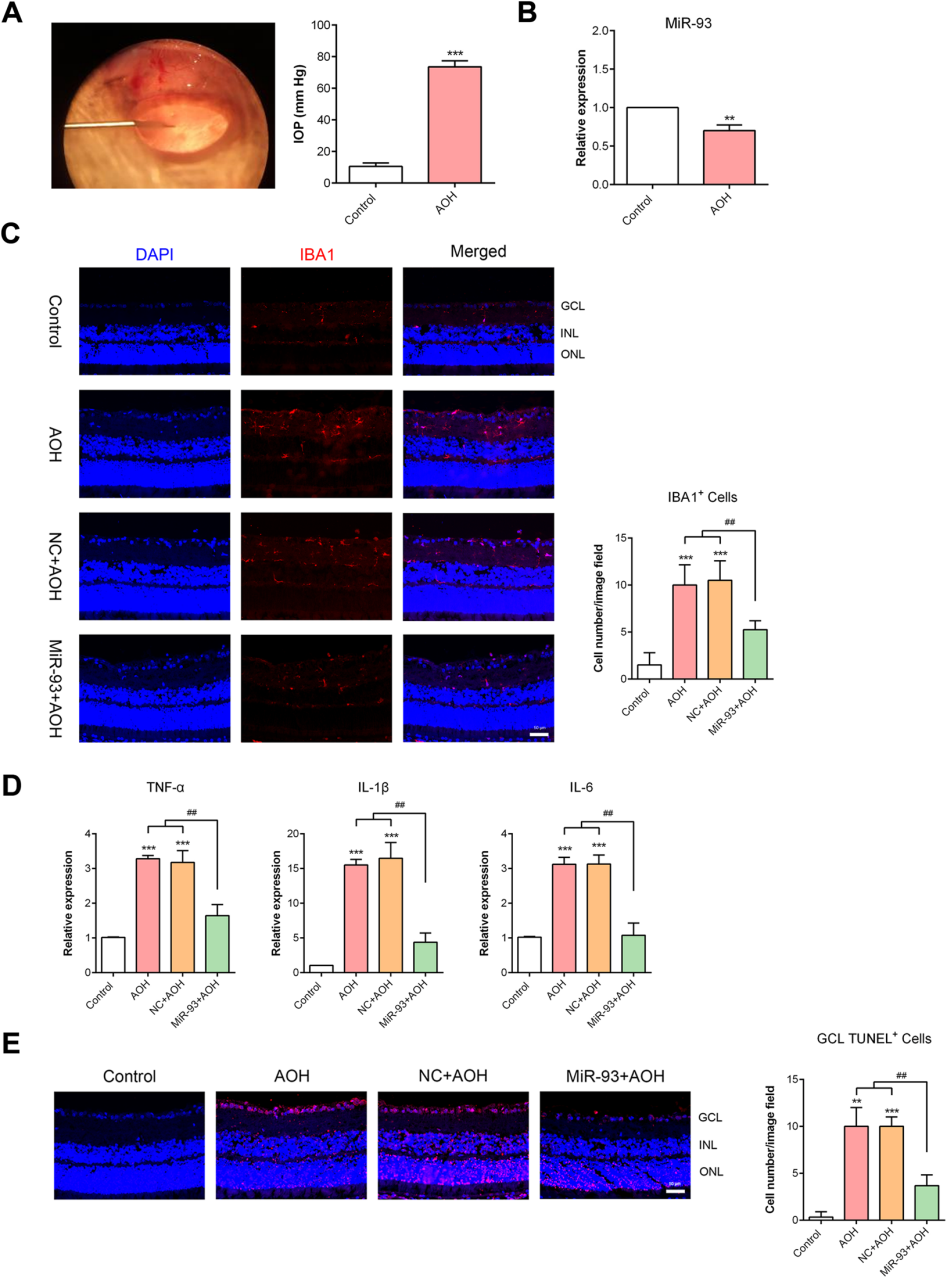
MiR-93 regulated retina microglial activation and neuronal apoptosis in AOH injury. **A** Rat acute ocular hypertension model. As the infusion needle was placed into the anterior chamber of the rat eye, IOP was rapidly elevated and maintained for an hour. **B** The miR-93 level was significantly decreased in AOH retinae versus controls. **C** Retinal microglial reaction in the AOH model. More microglia cells co-labelled with IBA1 (red) and DAPI (blue) were shown in retinae of AOH model, changed cell shape and migrated to the ganglion cell layer. MiR-93 upregulation suppressed microglial activation, as shown by fewer cells compared to the number in AOH retinae, while NC injection alone did not have any obvious influence. **D** QRT-PCR showed much higher cytokines expression in the retinae exposed to AOH injury. However, miR-93 overexpression in the eyes significantly ameliorated retinal neuroinflammation compared with that in AOH and NC+ AOH eyes. **E** As measured by TUNEL staining, the apoptosis of retinal cells was noticeably increased in response to AOH injury. Intravitreal miR-93 rescued retinal GCL cells from AOH-induced damage. Compared with the control: ^**^P < 0.01, ^***^P < 0.001. Compared with the AOH and NC+ AOH group: ^##^*P* < 0.01. *n* = 3 retinae. NC + AOH, agomir negative control + AOH; miR-93 + AOH, miR-93 agomir + AOH; GCL ganglion cell layer, INL inner nuclear layer, ONL outer nuclear layer.

We induced AOH injury in rats, and the IOP was elevated from 10 mmHg to approximately 75 mmHg for 1 h (Fig. 1A). The neuroretina was dissected for further detection. Thirty-one differentially expressed miRNAs were detected in AOH retinae versus control retinae by miRNA microarray analysis^18^, and the change in miR-93 was confirmed by qRT-PCR (Fig. 1B). In the control retinae, very few IBA1^+^ cells were observed and mostly located in the inner nuclear and inner plexiform layers. After AOH injury, the density of microglial cells was increased, and many cells migrated to the ganglion cell layer (GCL). In addition, the microglial morphology changed from a thin cell body with branched extensions to a large, amoeboid cell body with short branches (Fig. 1C). Furthermore, AOH injury increased multiple inflammatory cytokines, namely, TNF-α, IL-1β and IL-6, in the retina and markedly induced retinal cell apoptosis (Fig. 1D, E). These results illustrated that AOH caused miR-93 deficiency, thus playing a potential pathogenic role in controlling retinal microglial overactivation and cytokine overproduction.

### MiR-93 overexpression protected retinae against AOH by regulating the microglial reaction

To explore the biological effect of miR-93 in vivo, we injected miR-93 agomir and NC into the vitreous body before AOH induction. The overexpression of miR-93 clearly inhibited microglial activation by ~50% compared to that in AOH eyes (Fig. 1C). qRT-PCR showed that the mRNA levels of cytokines TNF-α, IL-1β and IL-6 were significantly decreased in miR-93-treated AOH retinae (Fig. 1D). Moreover, miR-93 substantially reduced the number of TUNEL^+^ cells in the retina, suggesting a neuroprotective effect (Fig. 1E). But NC injection did not induce any obvious change. Taken together, these findings suggested that miR-93 regulated the neurotoxic microglial overreaction, relieved neuro-inflammation and played a protective role in the AOH model.

### MiR-93 targets STAT3 gene and thus influenced microglial activation

**Fig. 2:**
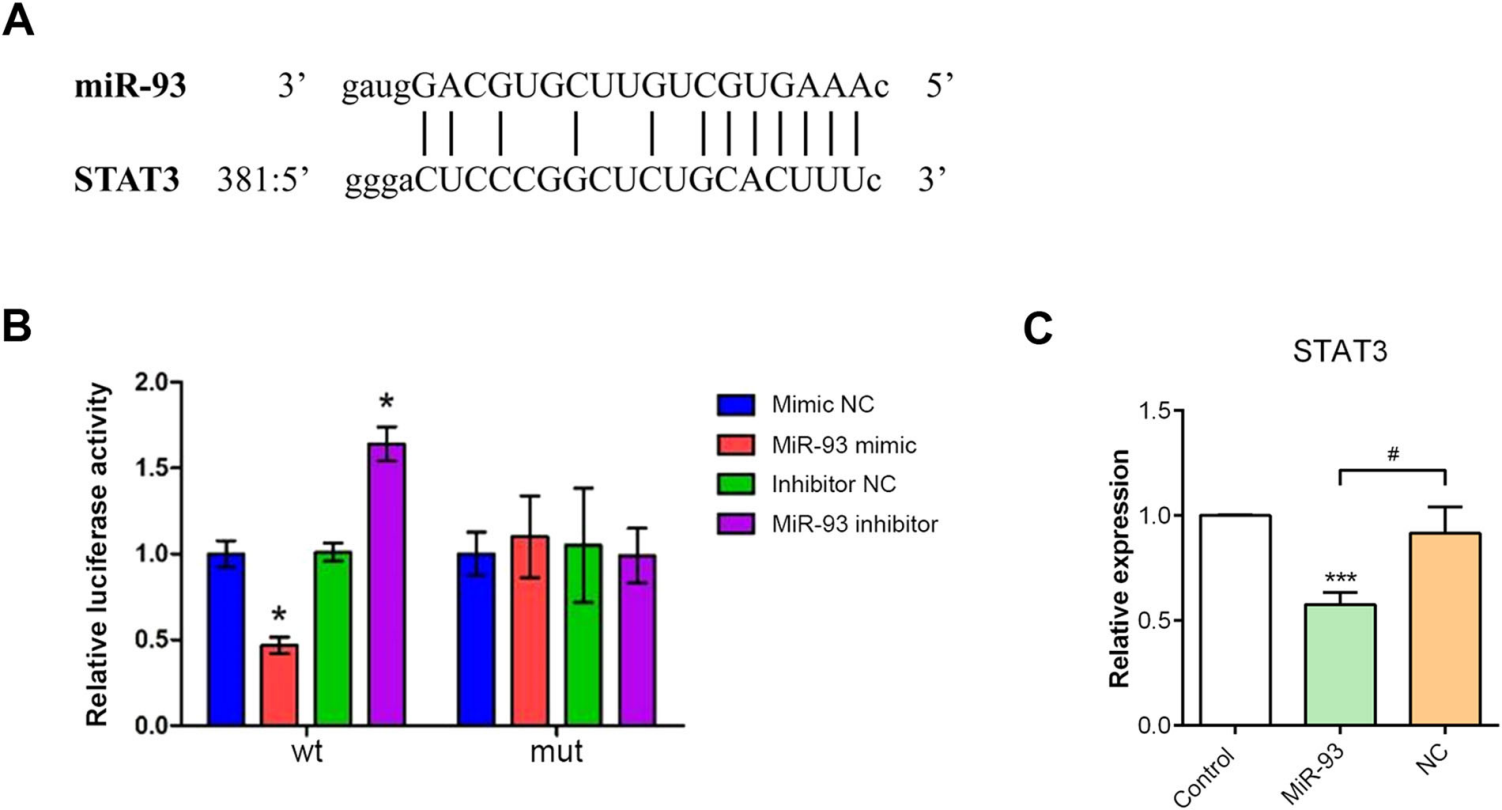
MiR-93 targeted the STAT3 gene. **A** Bioinformatics analysis predicted interactions between miR-93 and STAT3. The 3′-UTR of STAT3 contains a conserved binding site for miR-93. **B** The miR-93-dependent change in luciferase activity was abolished after the introduction of mutations in the STAT3 binding site, indicating that STAT3 gene is a target of miR-93. **C** As measured by qRT-PCR, miR-93 mimic transfection significantly decreased the STAT3 mRNA level in primary retinal microglial cells. Compared with the NC: ^*^P < 0.05. Compared with the control: ^***^P < 0.001. Compared with the miR-93 transfection group: ^#^*P* < 0.05. *n* = 3 independent cultures. NC negative control, wt wild type, mut mutant type.

**Fig. 3:**
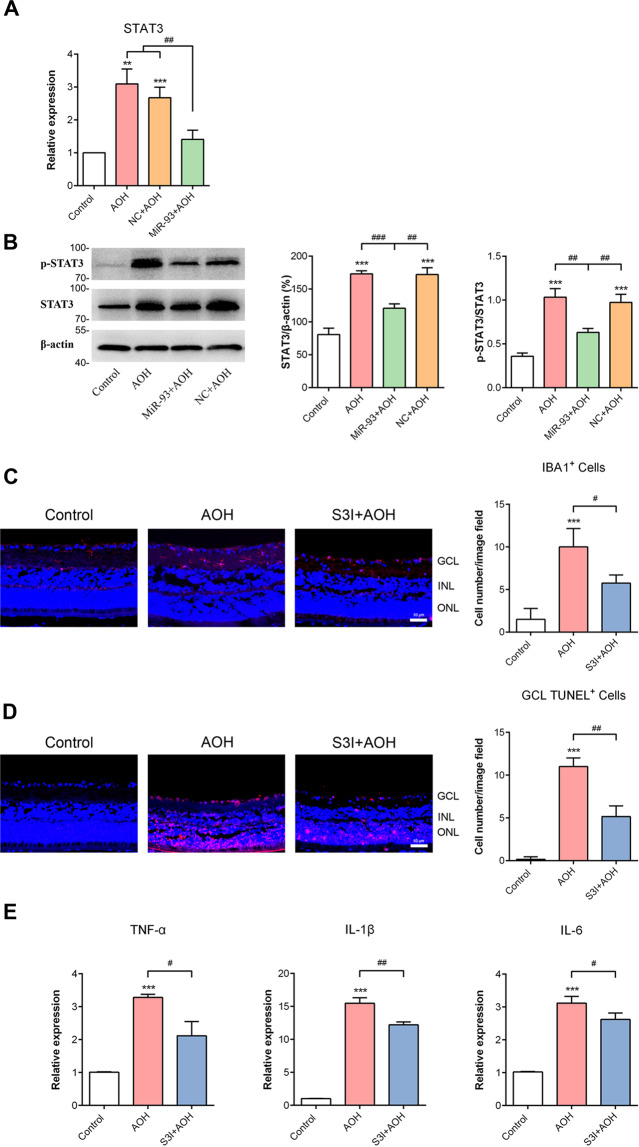
Overexpression of miR-93 suppressed STAT3 expression and STAT3 inhibitor influenced microglial activation in AOH model. **A** QRT-PCR showed that the upregulated STAT3 level observed after AOH was significantly decreased by miR-93 agomir but not by NC treatment. **B** The relatively high expressions of STAT3 and p-STAT3 protein observed in retinae after AOH was reduced by miR-93 agomir injection, as detected by western blotting. However, intravitreal injection of NC had little influence compared to AOH. **C** Treatment with a STAT3 inhibitor, S3I–201 (S3I), could inhibit microglial activation compared to AOH retinae. **D** S3I treatment reduced retinal GCL apoptotic cells after AOH injury. **E** S3I treatment inhibited the AOH-mediated overproduction of TNF-α, IL-1β, and IL-6. Compared with the control: ^**^P < 0.01, ^***^P < 0.001. Compared with the AOH or NC group: ^#^*P* < 0.05, ^##^*P* < 0.01, ^###^*P* < 0.001. *n* = 3 retinae. NC negative control, miR-93 miR-93 agomir, S3I + AOH, S3I–201 + AOH.

To further investigate the potential target by which miR-93 regulates microglial responses, we performed a bioinformatics analysis (www.targetscan.org/, mirwalk.umm.uni-heidelberg.de/ and mirdb.org/) and found that the STAT3 3′-UTR contains a conserved binding site for miR-93 (Fig. 2A)^21^. Next, we performed a dual-luciferase reporter assay and verified that STAT3 is a target gene of miR-93 in microglial cells. As shown in Fig. 2B, miR-93 mimic and STAT3 combined and greatly decreased the luciferase activity, while miR-93 inhibitor showed the opposite effect. The mutation of the binding site inhibited the luciferase activity change. Next, we directly examined the effect of miR-93 mimic transfection on STAT3 expression in primary retinal microglial cells by qRT-PCR. Results showed that miR-93 overexpression significantly decreased the STAT3 mRNA level in vitro (Fig. 2C). In vivo, AOH injury increased the mRNA level of STAT3 by approximately three-fold compared with that in the controls. However, target STAT3 mRNA was significantly decreased in miR-93-treated AOH retinae (Fig. 3A). The western blotting results confirmed that the increase in STAT3 protein expression after AOH was suppressed by miR-93, but not by NC injection (Fig. 3B). In addition, we investigated whether the phosphorylated status of STAT3 exposed to AOH injury could be affected by miR-93 injection. In both AOH and NC + AOH groups, the levels of phospho-STAT3 were markedly increased compared with the control group. However, miR-93 application significantly blocked phopsho-STAT3 expression in the retina (Fig. 3B). Together, these results suggested that miR-93 specifically affects STAT3 expression and activation.

To investigate whether STAT3 is implicated in regulating retinal microglial cells, we tested the effects of S3I-201, a STAT3 inhibitor, in AOH rat model. The results showed that S3I-201 injection significantly reduced the number of activated retinal microglial cells (Fig. 3C) and apoptotic cells in the GCL (Fig. 3D). S3I–201 treatment also attenuated the overproduction of inflammatory cytokines induced by AOH (Fig. 3E). Based on the above, miR-93 could function as a novel and effective STAT3 inhibitor and thus influenced microglial activation.

### OGD1 h/R24 h markedly induced primary retinal microglial cell activation

**Fig. 4:**
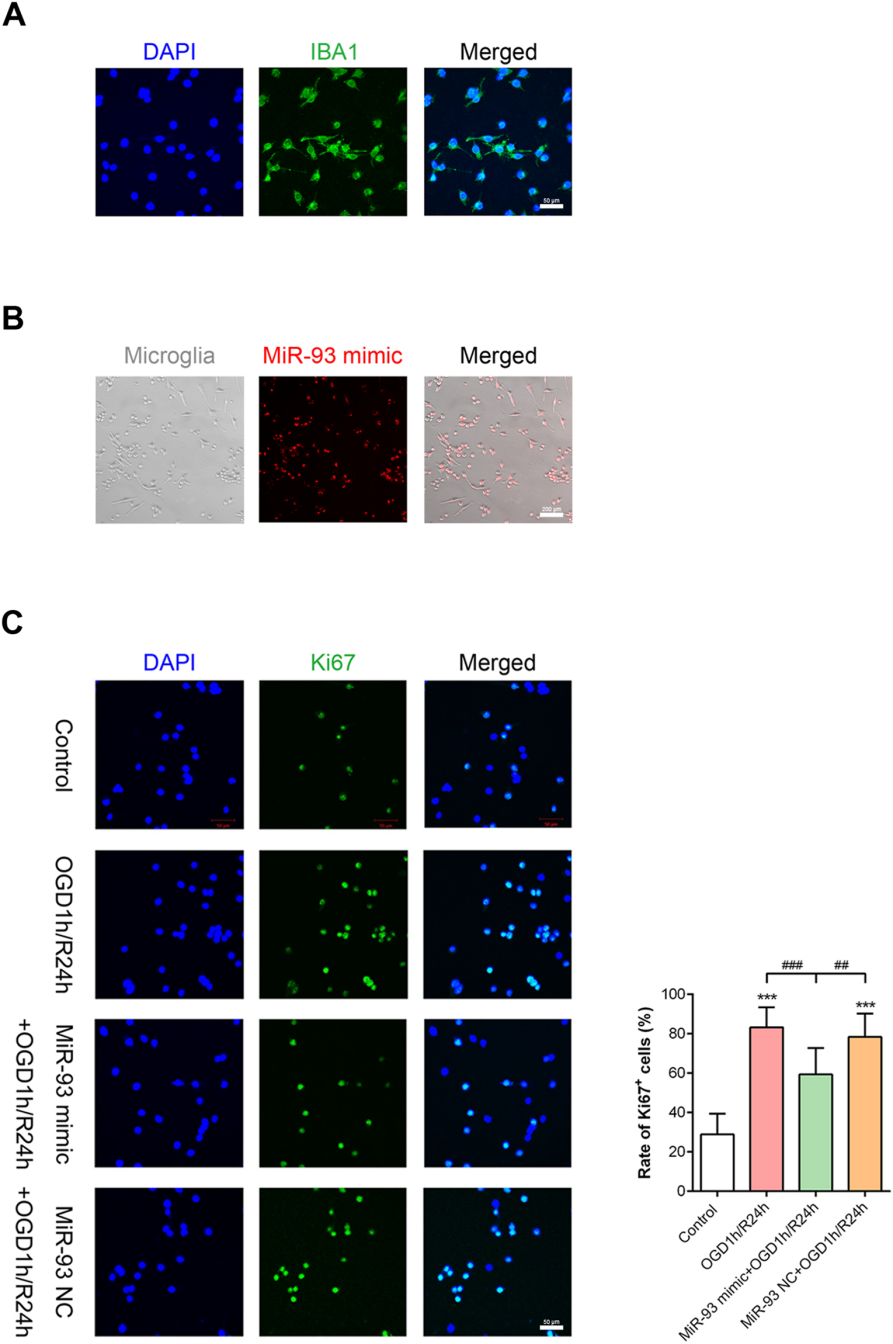
Overexpression of miR-93 inhibited microglial proliferation after OGD1 h/R24 h. **A** Cultured cells were microglia, as identified by IBA1^+^ (green) and DAPI^+^ (blue) staining. **B** MiR-93 mimic (red) was successfully transfected into more than 99% microglial cells. **C** As measured by immunocytochemistry for Ki67 (green), the marked OGD-stimulated microglial proliferation was inhibited by miR-93 transfection; this was evidenced by a reduction in the proportion of Ki67^+^ cells. But NC transfection had little effect. Compared with the control: ^***^P < 0.001. Compared with the OGD1h/R24h and miR-93 NC + OGD1 h/R24 h group: ^##^*P* < 0.01, ^###^*P* < 0.001. *n* = 3 independent cultures. OGD1 h/R24 h, oxygen–glucose deprivation for 1 h and reperfusion for 24 h; NC mimic negative control.

**Fig. 5:**
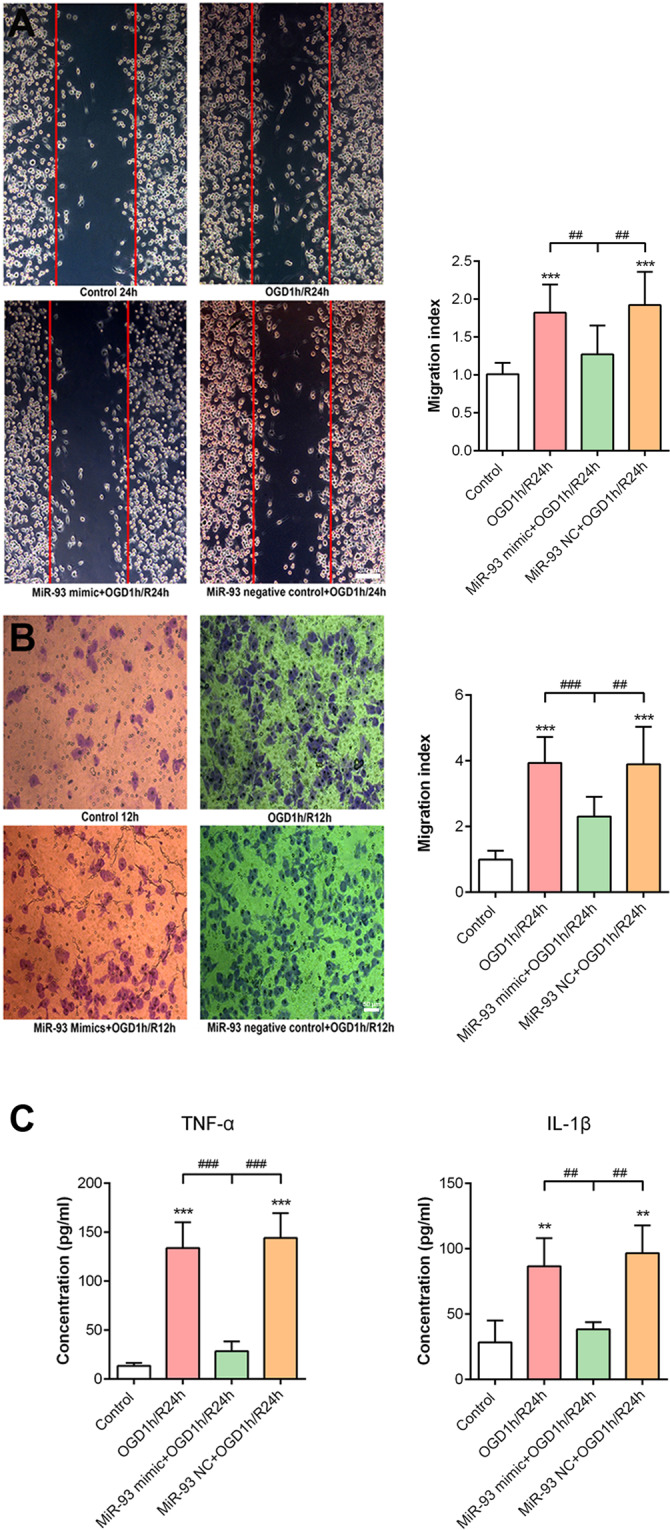
Overexpression of miR-93 inhibited microglial migration and cytokine production after OGD1 h/R24 h. **A**, **B** The scratch test and transwell assay showed that microglial migration indices were markedly increased by OGD1 h/R24 h stimulus and were effectively reduced by miR-93 overexpression but not by NC transfection. **C** MiR-93 overexpression inhibited the OGD1 h/R24 h-mediated overproduction of TNF-α and IL-1β by microglia cells, as measured by ELISA. Compared with the control: ^**^P < 0.01, ^***^P < 0.001. Compared with the OGD1h/R24h and miR-93 NC + OGD1h/R24h group: ^##^*P* < 0.01, ^###^*P* < 0.001. *n* = 3 independent cultures. OGD1h/R24h, oxygen–glucose deprivation for 1 h and reperfusion for 24 h; NC, mimic negative control.

Next, we focused on the effect of miR-93 on retinal microglial cells and transfected miR-93 mimic into OGD1 h/R24 h-exposed primary microglial cells (Fig. 4A, B). Immunocytochemistry showed that almost all cells harvested after separation by shaking were IBA1^+^ microglial cells (Fig. 4A). In the OGD1 h/R24 h group, the number of Ki67^+^ cells was increased compared with that in the controls, suggesting that microglia markedly proliferated (Fig. 4C). In addition, the scratch wound assay and transwell assay both illustrated that the migration of microglial cells was significantly improved after OGD1 h/R24 h (Fig. 5A, B).

### MiR-93 overexpression regulated microglial cell activation and inhibited inflammation in vitro

To improve the miR-93 level in cultured microglial cells, miR-93 mimic was transfected into cells, and this transfection was highly efficient (Fig. 4B). The overexpression of miR-93 significantly inhibited ~25% of the proliferation (Fig. 4C) and ~40% of the migration of microglial cells (Fig. 5A, B) observed after OGD1 h/R24 h, while transfection with miR-93 NC lost the potency. In addition, the levels of inflammatory cytokines including TNF-α and IL-1β, which were produced at high levels by activated microglial cells, were substantially suppressed by miR-93 transfection (Fig. 5C). These results indicated that miR-93 regulated microglial cell activation and inhibited inflammatory factors released in vitro.

## Discussion

Resident microglial cells in the retina can be regarded as immunological watchdogs that rapidly respond to various insults by morphologically and functionally transforming into reactive phagocytes^22^. In neurodegenerative diseases, activated microglial phenotypes are complicated and can be neurotoxic or neuroprotective in response to specific stimulus and model^23,24^. Reactive microglial cells can secrete neurotrophic factors^25^, remove necrotic tissue and promote tissue repair^26^. However, once they get out of control, the overactivation of microglial inflammatory responses can aggravate the neural injury and ultimately cause diseases^27^. Studies have suggested that such microglial activation in the retina occurs in human eyes with glaucoma and in animals with experimental glaucoma^28^. In the AOH model, sharp IOP elevation would induce microglial number increase, morphology changes, distribution migration and inflammatory factors release, mainly performing neurotoxic and pro-inflammatory functions^2,3,18,29^. It subsequently leads to a progressive loss of RGCs and their axons and irreversible vision loss. Regulating microglial cells to broadly alleviate retinal neuroinflammation may thus be an important strategy for neuroprotection. Some candidate compounds, such as minocycline, erythropoietin and triamcinolone acetonide, have been reported to be effective in retinal microglial modulation in rodents; however, their use is associated with a variety of side effects^3,30,31^.

Since the discovery of miRNAs, they have emerged as key regulators of biological processes, including cell proliferation, differentiation, homoeostasis and death. Regulating miRNAs has become an attractive therapeutic approach for an increasing number of diseases, such as cancer and CNS disorders^32^. Some miRNAs, such as miR-155 and miR-124, have been known to be involved in microglia-mediated immune responses in the CNS^33^. In our previous study, the concentration of miR-93 was shown to be significantly reduced in the aqueous humour of glaucoma patients and in the retinae of rodents with experimental glaucoma. However, little is known about the function and mechanism of miR-93 in the process of glaucomatous optic nerve injury.

**Fig. 6:**
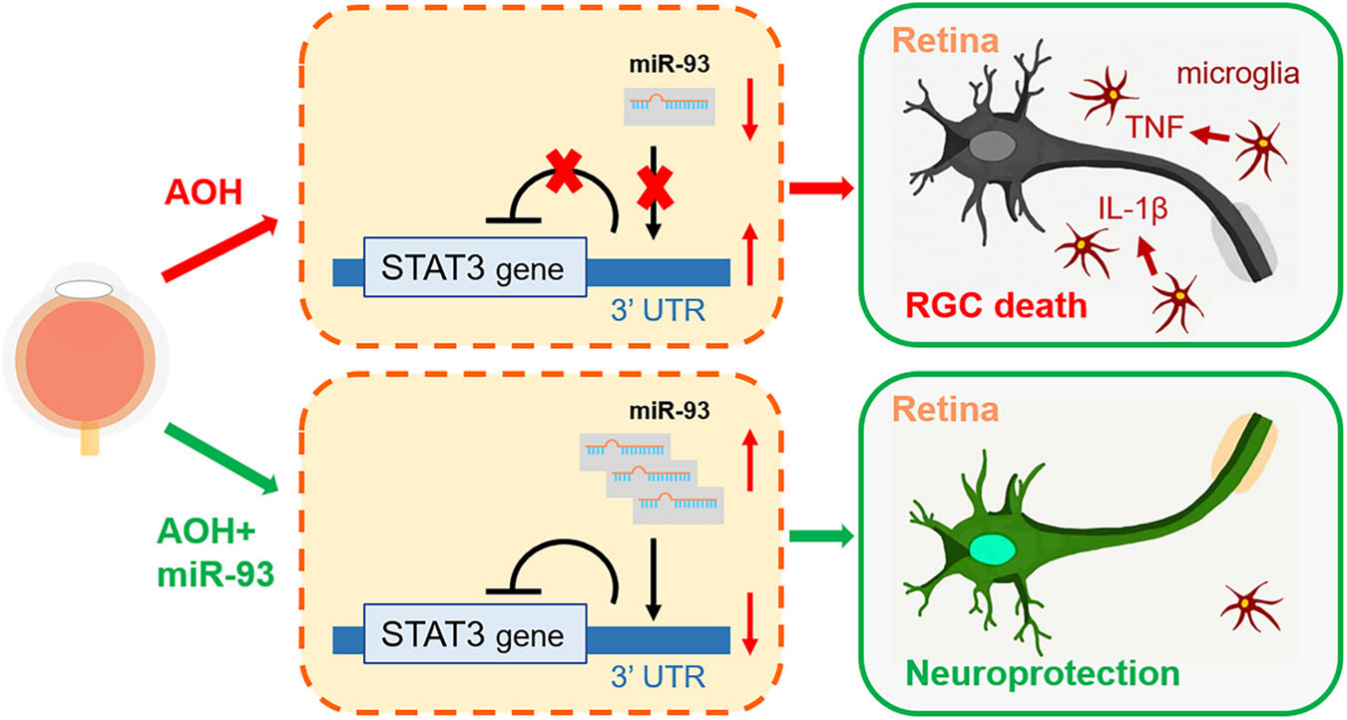
Graphical summary of the miR-93/STAT3 pathway in AOH injury. In AOH eyes, downregulated miR-93 releases the repression of downstream target genes of STAT3. Retinal microglial cells markedly activate and overproduce inflammatory cytokines, ultimately leading to retinal ganglion cell (RGC) death. Supplementation with miR-93 can represses STAT3 by binding to the 3′UTR of STAT3, regulating microglia-mediated neuro-inflammation and protecting RGCs against AOH. Thus, miR-93 may serve as a novel target for neuroprotective therapy in glaucoma.

Previously, miR-93 has been studied in many diseases, including cancer, diabetes, and cardiac and cerebral ischaemic injury. Studies have shown an important role for miR-93 in inhibiting microglia-mediated neuroinflammation in the nervous system. Spinal cord miR-93 was significantly decreased in rat models of chronic neuropathic pain, while the overexpression of miR-93 relieved pain and suppressed the expression of inflammatory cytokines including IL-1β, TNF-α and IL-6 in vivo^34^. MiR-93 levels were reduced in the blood of acute stroke patients and in the brains of ischaemia-reperfusion injury mice^35,36^. The application of miR-93 could alleviate the injury and inflammatory responses by binding with interleukin-1 receptor-associated kinase 4^36^. In vitro, miR-93 application decreased the OGD-induced proliferation of BV2 microglial cells^35^. It remains unclear whether miR-93 can regulate retinal microglial cells and influence neuroinflammation in glaucoma. In the present study, we observed a role for miR-93 in inhibiting retinal microglia overactivation and inflammatory responses. The overexpression of miR-93 significantly attenuated the apoptosis of RGCs in the AOH model. We also found that STAT3 is the target of miR-93 (Fig. 6). Our results suggested that miR-93 may represent a potential therapeutic target for controlling neuroinflammation in glaucoma. In addition, an investigation reported that miR-93 was downregulated in *N*-methyl-d-aspartate-treated glaucoma rats and RGCs in vitro. The upregulation of miR-93, which targets phosphatase and tensin homologue, suppressed the autophagy of RGCs through the AKT/mTOR pathway^37^.

Through dual-luciferase reporter assays and experimental intervention in rats, we identified STAT3 as a target gene of miR-93. STAT3 is an important transcription factor that regulates cytokine-dependent inflammation and immunity^38^. In the Janus kinase (JAK)-STATs pathway, STAT3 is phosphorylated by activated cytokine receptors and forms stable homodimers or heterodimers with other STAT proteins. Activated STATs respond to cytokines and establish a feedforward inflammation loop in the injured microenvironment. The JAK/STATs system has been described to be significantly activated in spinal and brains microglial cells, contributing to inflammatory gene expression^39,40^. Western blot analysis has shown STAT3 activation after different injuries in retinal tissue, and inhibition of the JAK/STATs pathway led to anti-inflammatory effects and protected RGCs^41–44^. A previous study reported that a STAT3 inhibitor WP1066 could effectively inhibit the migration of microglial cells and reduce TNF-α production^45^. Moreover, retrobulbar injection of AG490, an inhibitor of STAT3, protected RGCs in rat AOH model^46^. In the present study, we found that the overexpression of miR-93 significantly reduced the expression of the target gene STAT3 and its phosphorylation, inhibited microglial overactivation and reduced the release of inflammatory cytokines, protecting RGCs. Our results are consistent with previously reported findings, suggesting that specifically targeting STAT3 with miR-93 may effectively break the inflammatory loop and relieve secondary injury in glaucoma.

There are some limitations to this study. First, we focused on microglial cells, but other retinal cell types may also be influenced by miR-93. Although we observed the effect of miR-93 in cultured isolated primary retinal microglial cells, the non-specific effects of intraocular administration might have contributed to the observations made in the in vivo experiments. Second, miR-93 can likely regulate a multitude of target genes. We examined whether miR-93 specifically suppressed the expression of STAT3, while other potential targets may include MAPK9, MAP3K12 and caspase 3, which are all likely to also be involved in the miR-93 pathway. Third, since our findings were based on cell culture investigations and experimental animal studies, they cannot directly be generalised to patients. Since current clinical trials are testing miRNA-based treatments for some cancers, future studies may address the question of whether regulating miRNAs, including miR-93, may be of clinical benefit for treating ocular diseases, including glaucoma.

In conclusion, our study reported a neuroprotective effect of miR-93 in retinal AOH injury. MiR-93 negatively regulated microglia-mediated neuro-inflammatory responses and protected RGCs by targeting STAT3. Our results suggest that miR-93 may serve as a novel target for future neuroprotective treatment for glaucoma.
